# Shared N417-Dependent Epitope on the SARS-CoV-2 Omicron, Beta, and Delta Plus Variants

**DOI:** 10.1128/jvi.00558-22

**Published:** 2022-07-13

**Authors:** Thandeka Moyo-Gwete, Mashudu Madzivhandila, Nonhlanhla N. Mkhize, Prudence Kgagudi, Frances Ayres, Bronwen E. Lambson, Nelia P. Manamela, Simone I. Richardson, Zanele Makhado, Mieke A. van der Mescht, Zelda de Beer, Talita Roma de Villiers, Wendy A. Burgers, Ntobeko A. B. Ntusi, Theresa Rossouw, Veronica Ueckermann, Michael T. Boswell, Penny L. Moore

**Affiliations:** a National Institute for Communicable Diseases of the National Health Laboratory Service, Johannesburg, South Africa; b MRC Antibody Immunity Research Unit, School of Pathology, Faculty of Health Sciences, University of the Witwatersrand, Johannesburg, South Africa; c Department of Immunology, Faculty of Health Sciences, University of Pretoria, Pretoria, South Africa; d Tshwane District Hospital, Pretoria, South Africa; e Institute of Infectious Disease and Molecular Medicine, Division of Medical Virology, Department of Pathology, University of Cape Town, Cape Town, South Africa; f Wellcome Centre for Infectious Diseases Research in Africa, University of Cape Town, Cape Town, South Africa; g Division of Cardiology, Department of Medicine, University of Cape Town and Groote Schuur Hospital, Cape Town, South Africa; h Cape Heart Institute, Faculty of Health Sciences, University of Cape Town, Cape Town, South Africa; i Division for Infectious Diseases, Department of Internal Medicine, Steve Biko Academic Hospital and University of Pretoria, Pretoria, South Africa; j Centre for the AIDS Programme of Research in South Africa (CAPRISA), Durban, South Africa; Loyola University Chicago

**Keywords:** antibody cross-reactivity, antibody isolation, SARS-CoV-2, variants

## Abstract

As severe acute respiratory syndrome coronavirus 2 (SARS-CoV-2) continues to evolve, several variants of concern (VOCs) have arisen which are defined by multiple mutations in their spike proteins. These VOCs have shown variable escape from antibody responses and have been shown to trigger qualitatively different antibody responses during infection. By studying plasma from individuals infected with either the original D614G, Beta, or Delta variants, we showed that the Beta and Delta variants elicit antibody responses that are overall more cross-reactive than those triggered by D614G. Patterns of cross-reactivity varied, and the Beta and Delta variants did not elicit cross-reactive responses to each other. However, Beta-elicited plasma was highly cross-reactive against Delta Plus (Delta+), which differs from Delta by a single K417N mutation in the receptor binding domain, suggesting that the plasma response targets the N417 residue. To probe this further, we isolated monoclonal antibodies from a Beta-infected individual with plasma responses against Beta, Delta+, and Omicron, which all possess the N417 residue. We isolated an N417-dependent antibody, 084-7D, which showed similar neutralization breadth to the plasma. The 084-7D MAb utilized the IGHV3-23*01 germ line gene and had somatic hypermutations similar to those of previously described public antibodies which target the 417 residue. Thus, we have identified a novel antibody which targets a shared epitope found on three distinct VOCs, enabling their cross-neutralization. Understanding antibodies targeting escape mutations, such as K417N, which repeatedly emerge through convergent evolution in SARS-CoV-2 variants, may aid in the development of next-generation antibody therapeutics and vaccines.

**IMPORTANCE** The evolution of SARS-CoV-2 has resulted in variants of concern (VOCs) with distinct spike mutations conferring various immune escape profiles. These variable mutations also influence the cross-reactivity of the antibody response mounted by individuals infected with each of these variants. This study sought to understand the antibody responses elicited by different SARS-CoV-2 variants and to define shared epitopes. We show that Beta and Delta infections resulted in antibody responses that were more cross-reactive than the original D614G variant, but they had differing patterns of cross-reactivity. We further isolated an antibody from Beta infection which targeted the N417 site, enabling cross-neutralization of Beta, Delta+, and Omicron, all of which possess this residue. The discovery of antibodies which target escape mutations common to multiple variants highlights conserved epitopes to target in future vaccines and therapeutics.

## INTRODUCTION

Since the emergence of severe acute respiratory syndrome coronavirus 2 (SARS-CoV-2) in 2019, several variants of concern (VOCs) and interest (VOIs) have emerged. Many of these variants contain mutations in the spike protein that confer escape from neutralizing antibodies (1). The first neutralization-resistant VOC identified, Beta, contained receptor binding domain (RBD) mutations at positions K417N and E484K, which confer escape from class I and class II neutralization antibodies, respectively (2, 3). These mutations have since been observed in multiple variants. One such example is the charge-altering E484K/Q/A mutation, which occurs in several variants, including Gamma, the Delta lineage, C.1.2, and A.VOI.V2 (1, 4–6). The E484K mutation is also present in Omicron (first identified in South Africa), which is the most neutralization-resistant variant identified to date, evading both convalescent-phase and vaccine-elicited antibody responses (5, 7–9).

Similarly, the K417N mutation found in the Beta variant has also emerged in other VOCs and VOIs throughout the world. This mutation results in a change from a positively charged residue (lysine [K]) to an uncharged asparagine (N) residue, a difference large enough to prevent binding of antibodies dependent on this epitope (3). This substitution results in escape from class I neutralizing antibodies such as the therapeutic monoclonal antibody (MAb) etesevimab (10, 11). The K417N mutation also occurs in a sublineage of Delta, called Delta Plus (Delta+), and in the Omicron variant (BA.1) and its sublineages (BA.2, BA.3, BA.4, and BA5) (12), indicating that an antibody that targets this site may cross-neutralize these variants.

The high levels of convergent evolution observed globally between variants indicates that at a population level, neutralizing antibodies target similar regions of the SARS-CoV-2 spike protein (13). This is supported by numerous MAb isolation studies which identified the RBD as the major target of the neutralizing antibody response, regardless of the infecting variant or type of vaccination (14–18). However, there is substantial evidence that the antibody response toward the RBD differs in fine specificity depending on which variant triggered the infection. The original (D614G) variant triggers antibodies with very low levels of cross-reactivity to the Beta variant (3). Responses elicited by the Beta variant, however, exhibit higher levels of cross-reactivity toward the D614G and the Gamma variant (19) but not against the Delta variant (20, 21). Similarly, Delta-elicited responses have low cross-reactivity toward the Beta variant (16, 22). It has therefore been suggested that the Beta and Delta variants took divergent evolutionary pathways toward distinct groupings or “serotypes,” with the D614G original variant being serologically closer to Delta than Beta (20). The Omicron variant elicits predominantly Omicron-specific antibodies, with poor cross-reactivity against Delta, C.1.2, and D614G, and a dramatic 31-fold reduction in activity against Beta (23). Similar findings have been reported for common-cold coronaviruses (24). Defining the fine specificity of neutralizing antibodies elicited by the different variants is therefore important to determine whether there are common responses targeting divergent variants.

In this study, we compared the cross-reactivities of plasma responses during three separate SARS-CoV-2 waves in South Africa. Each epidemic wave in South Africa was dominated by a distinct variant: first D614G and then Beta, followed by Delta. We first assessed the neutralizing antibody responses elicited by each of the three variants against a panel of circulating VOCs and SARS-CoV-1. We show that Beta and Delta infections elicited more cross-reactive responses than the D614G variant, though the patterns of cross-reactivity were different. Furthermore, Beta-elicited plasma potently neutralized the Delta+ variant compared to the Delta variant, suggesting that these cross-reactive antibodies may target the N417 residue, as this is the only spike mutation that differentiates these two variants. To confirm this, we isolated and characterized a MAb from a Beta-infected individual who showed plasma neutralization of Beta, Delta+, and Omicron. Epitope mapping revealed that this MAb, 084-7D, recapitulated much of the plasma breadth and was heavily dependent on a shared N417 epitope. The discovery of cross-reactive MAbs may aid in the development of universal SARS-CoV-2 therapeutic antibodies and inform the design of booster vaccines.

## RESULTS

### Neutralizing responses triggered by variants of concern possess differential patterns of cross-reactivity.

**FIG 1:**
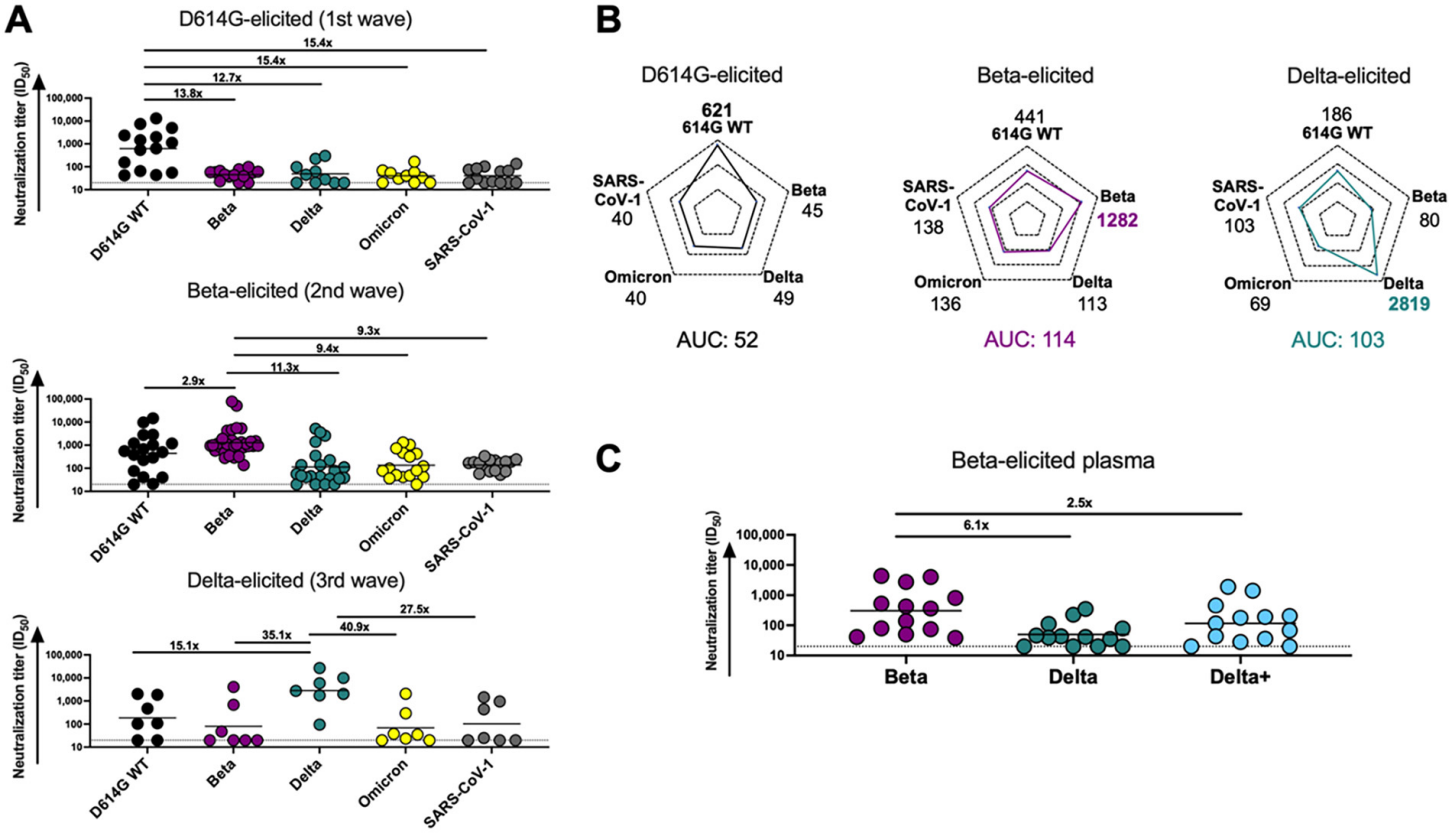
Comparison of plasma cross-reactivities elicited by three distinct SARS-CoV-2 variants. (A) Plasma from D614G, Beta, and Delta infections during three distinct SARS-CoV-2 waves were tested for neutralization breadth against a range of VOCs using a pseudovirus-based neutralization assay. Fold changes in neutralization are shown above each variant. Plasma neutralization titer is measured as an ID_50_ (reciprocal plasma dilution causing 50% reduction of infection). Black horizontal bars represent geometric means. The threshold of detection for the neutralization assay is an ID_50_ of >20. (B) Spider plots were derived from GMTs for plasma triggered by D614G, Beta, or Delta against multiple VOCs. The GMTs for each plasma set were normalized against titers to the autologous virus, and breadth was expressed as area under the curve. (C) Plasma from Beta-infected individuals was tested against the Delta and Delta+ variants. Fold changes in neutralization are shown above each variant. Plasma neutralization titer is measured as an ID_50_. Black horizontal bars represent geometric means. The threshold of detection for the neutralization assay is an ID_50_ of >20.

We first compared antibody responses in unvaccinated individuals infected by VOCs circulating in three distinct SARS-CoV-2 epidemiological waves in South Africa from May 2020 to July 2021. In each wave, sampling occurred at the time when each respective variant was responsible for at least 90% of infections, and sequencing in a subset of matched nasal swabs confirmed the infecting variant. We investigated how well plasma elicited by D614G, Beta, and Delta variants neutralized multiple VOCs, as well as SARS-CoV-1, using a pseudovirus neutralization assay (Fig. 1A and B). D614G-elicited plasma triggered robust neutralization of the matched D614G spike (autologous neutralization) but showed very low levels of cross-reactivity, with a 12- to 15-fold decrease in neutralization toward all SARS-CoV-2 VOCs tested and to SARS-CoV-1 (Fig. 1A). In contrast, as we previously reported, plasma from Beta infections was highly cross-reactive against the D614G variant (2.9-fold reduction) (19) but showed less cross-reactivity against other VOCs, with 11.3-fold, 9.4-fold, and 9.3-fold reductions against Delta, Omicron, and SARS-CoV-1, respectively (Fig. 1A). We also tested plasma from Delta-infected individuals and found that although autologous titers against the matched spike were very high, there was a 15- to 41-fold drop in neutralization potency across VOCs/VOIs and SARS-CoV-1. The higher level of cross-reactivity for the D614G variant (15-fold drop in potency compared to Beta [35-fold drop]) in Delta-elicited plasma is consistent with what others have reported (20, 22).

To get a measure of the degree and patterns of cross-reactivity for each variant, we compared the geometric mean titers (GMTs) of the plasma response elicited by the three variants toward various VOCs and SARS-CoV-1 using a spider plot (Fig. 1B). Each variant triggered the highest responses against their corresponding autologous virus as expected. Furthermore, the extent of autologous neutralization varied, with Delta triggering particularly high autologous responses (GMT, 2,819), compared to Beta (GMT, 1,282) and D614G (GMT, 621), perhaps a consequence of the high viral loads in Delta infections (25). We therefore calculated a single measure of breadth similar to previous studies, by assessing the cumulative area under the curve (AUC) value for all variants, normalized relative to the GMT of the autologous virus to account for differential potency of responses (Fig. 1B) (26). Beta- and Delta-elicited responses were both approximately 2-fold higher (AUCs, 114 and 103) than for D614G (AUC, 52) (Fig. 1B), indicating that the neutralizing responses triggered by these two variants were more cross-reactive than those elicited by the early circulating SARS-CoV-2 variant.

Although other sites, such as the RBD class III antibody binding site, have previously been implicated as targets of the Beta-elicited responses (15), the K417N mutation in the Beta RBD has also been shown to be a dominant target of Beta-elicited plasma (27, 28). To assess whether antibodies to this site accounted for the breadth in Beta-infected individuals, we tested Beta plasma against the Delta+ variant, which differs from Delta only at K417N, the same mutation as is also found in Beta (Fig. 1C). This mutation is the only shared mutation in the RBDs of the Beta and Delta+ lineages. Compared to the Beta response toward Delta, which showed a significant drop in potency (6.1-fold), there was only a 2.5-fold drop in potency against Delta+ (Fig. 1C). These data confirm the importance of the N417 residue as a target of Beta-elicited cross-reactive neutralizing antibody responses.

### Isolation and characterization of a SARS-CoV-2 cross-reactive, neutralizing monoclonal antibody from a Beta-infected individual.

**FIG 2:**
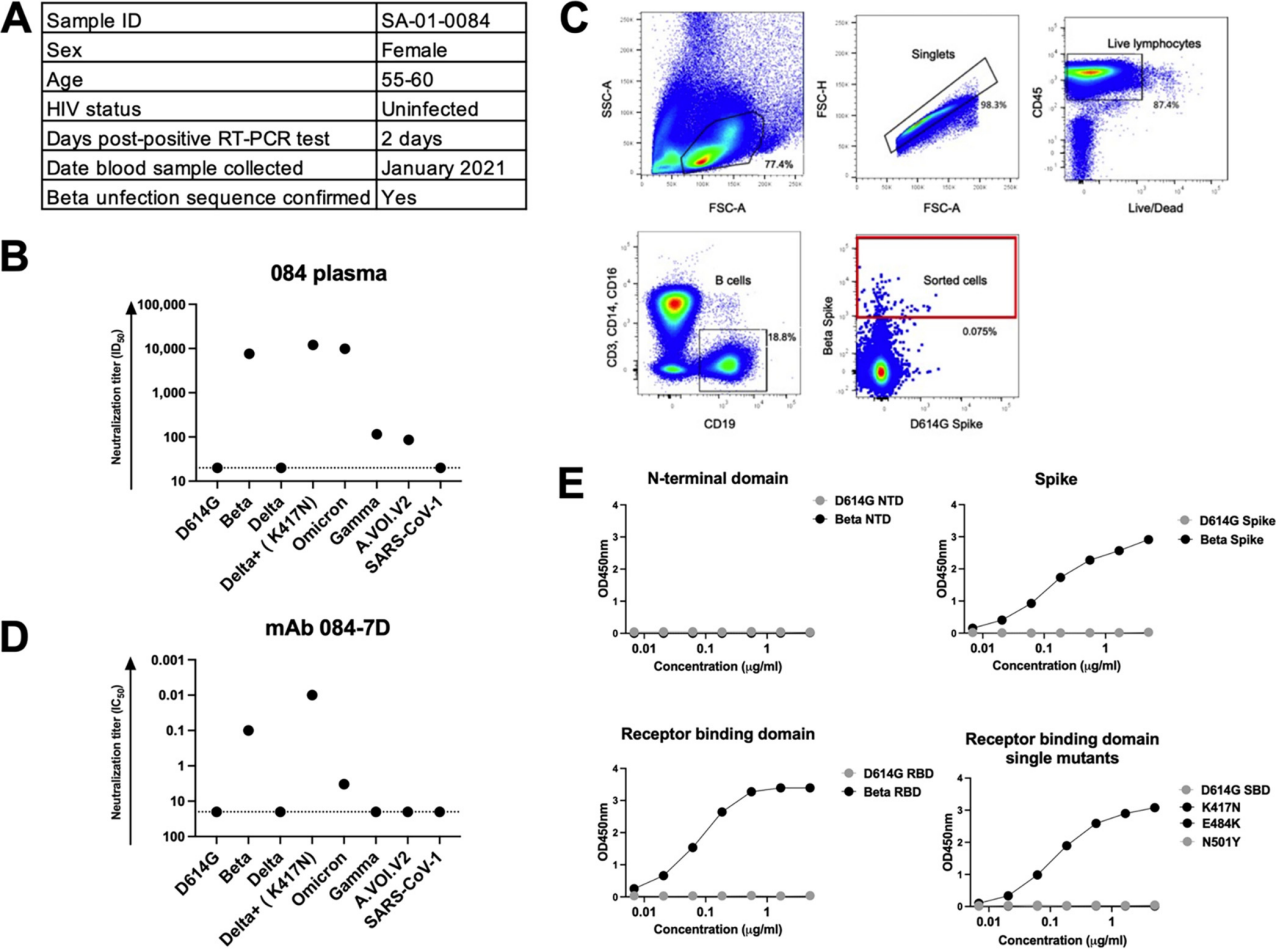
Characterization of plasma and an isolated monoclonal antibody from a Beta-infected individual. (A) A sequence-confirmed Beta-infected hospitalized individual, SA-01-0084 (084), was selected for this study. Plasma and peripheral blood mononuclear cells were collected 2 days post-positive PCR for further analysis. (B) Plasma from SA-01-0084 was tested for neutralization activity in a pseudovirus-based neutralization assay against a range of VOC/VOIs. Plasma neutralization titer is measured as an ID_50_. The threshold of detection for the neutralization assay is an ID_50_ of >20. (C) The gating strategy for the isolation of SARS-CoV-2 spike-specific B cells via single-cell sorting is shown. Each dot represents a cell, and the dots in the sorted cell red gate represent Beta spike-positive B cells that had CD3^−^, CD14^−^, CD16^−^, and CD19^+^ markers. These cells were single-cell sorted into 96-well plates and amplified through antibody gene-specific PCR. (D) MAb 084-7D was tested for neutralization activity in a pseudovirus-based neutralization assay against the same VOCs/VOIs as tested with the plasma. MAb neutralization was measured as a monoclonal antibody concentration causing 50% reduction of infection (IC_50_, in micrograms per milliliter). The threshold of detection for the neutralization assay is an IC_50_ of >20 μg/mL. (E) A SARS-CoV-2 ELISA was used to map the specificity of the 084-7D MAb. The MAb was tested against the D614G and Beta N-terminal domain, spike and RBD antigens, as well as SBD (subdomain 1) proteins mutated to include Beta-specific SBD single mutations, K417N, E484K, and N501Y. The starting concentration of the MAb was 5 μg/mL. The threshold of detection for the binding assay is an optical density at 450 nm (OD_450_) of >0.04. Lines for D614G SBD, E484K, and N501Y are all at negative and superimposed.

To assess whether the N417 residue was a target of Beta-elicited MAb responses, we sought to isolate MAbs from a Beta-infected individual who developed potent responses toward the Beta variant and was cross-reactive toward other VOCs. We previously recruited participant SA-01-0084 (084) into our cohort of hospitalized patients infected with the Beta variant (19). This participant was female, below the age of 60, and non-HIV infected and had their blood drawn 2 days after a positive SARS-CoV-2 reverse transcription-PCR (RT-PCR) result (Fig. 2A). Plasma from donor 084 potently neutralized Beta (reciprocal plasma dilution causing 50% reduction of infection [ID_50_] = 7,613) and Delta+ (ID_50_ = 12,083) pseudoviruses (Fig. 2B). The 084 plasma also potently neutralized Omicron (ID_50_ = 9,882). However, it did not neutralize the D614G, Delta, or SARS-CoV-1 pseudovirus and had weak neutralization against Gamma and A.VOI.V2 (ID_50_ < 120) (Fig. 2B). The high cross-reactivity of the plasma toward Delta+ was similar to what we observed with other Beta-elicited plasma (Fig. 1C).

We performed single-cell sorting of the participant’s peripheral blood mononuclear cells (PBMCs). Using Beta and D614G spike proteins as cell sorting baits, we sorted a total of 68 cells, with approximately 58 of the cells being Beta specific and the rest of the cells being doubly positive (Fig. 2C). We identified an IgG1 MAb, 084-7D, which bound to the Beta spike antigen (Fig. 2). We tested the functionality of MAb 084-7D by first examining its neutralizing activity against the same VOCs as we had tested the plasma against (Fig. 2D). The 084-7D MAb showed a very similar cross-reactivity pattern to the matched sera, exhibiting potent neutralization of Beta (concentration causing 50% reduction of infection [IC_50_] = 0.10 μg/mL) and Delta+ (IC_50_ = 0.01 μg/mL), though it showed slightly lower activity against Omicron (IC_50_ = 3.31 μg/mL) than was observed in the plasma. The MAb did not neutralize SARS-CoV-1 or the other VOCs/VOIs tested (Fig. 2D). These data indicate that this MAb recapitulated the dominant response in the plasma, though it did not entirely account for Omicron plasma neutralization.

### 084-7D MAb epitope mapping revealed spike target that is highly dependent on the N417 residue.

To map the spike target of the MAb, we conducted binding experiments using a SARS-CoV-2-specific enzyme-linked immunosorbent assay (ELISA). The antibody bound the full Beta spike but not the D614G spike as expected based on the neutralization assay results (Fig. 2E). No binding was observed for either the Beta or D614G N-terminal domain (NTD) proteins, but strong binding was detected toward the Beta RBD (Fig. 2E). We expressed single mutants representing the three RBD mutations found in the Beta variant—K417N, E484K and N501Y—in an SBD (subdomain 1) backbone and tested whether the antibody was able to recognize any of the Beta mutations. The 084-7D MAb bound to the K417N mutant but did not bind to the D614G SBD or E484K and N501Y single mutants (Fig. 2E). Overall, these data suggest that the 084-7D MAb is dependent on the N417 residue present in the Beta RBD.

### MAb 084-7D exhibits Fc effector functionality, particularly against the Beta variant.

**FIG 3:**
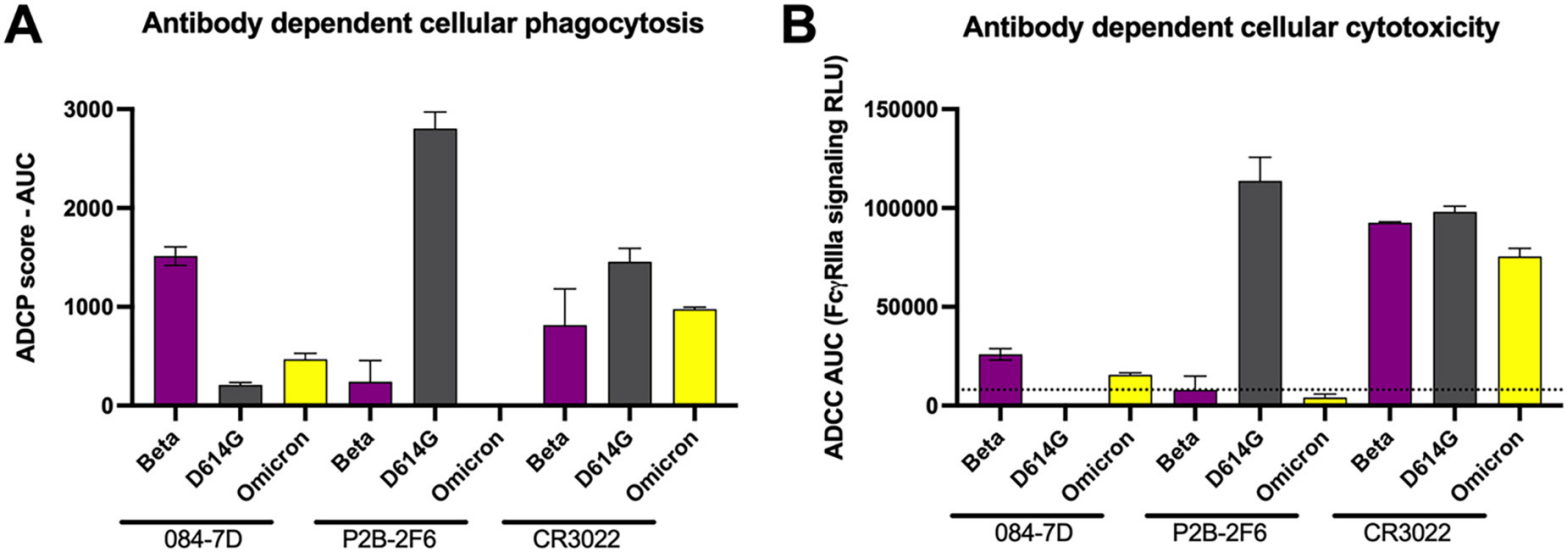
MAb 084-7D displayed antibody-dependent cellular phagocytosis (ADCP) and antibody-dependent cellular cytotoxicity (ADCC) with cross-reactivity toward Beta and Omicron. (A) ADCP activity of MAb 084-7D was measured using THP-1 phagocytosis assay and the phagocytosis score is shown as an area under the curve (AUC) measure. The threshold of detection for this assay is an AUC of  >0. (B) ADCC activity of MAb 094-7D was measured using an infectious ADCC assay. The percent killing activity is shown as an AUC measure. The threshold of detection for the ADCC assay is an AUC of >8,000. In both ADCP and ADCC assays, P2B-2F6 was used a positive control for D614G activity, CR3022 was used as a positive control for all variants, and palivizumab, a respiratory syncytial virus (RSV)-specific MAb, was used as a negative control. Experiments were conducted in duplicate, and bars represent the means with standard deviations of two experiments.

We have previously shown that in addition to neutralization, the Beta variant triggers cross-reactive Fc functionality compared to D614G (29). We therefore analyzed the ability of the 084-7D MAb to mediate antibody-dependent cellular phagocytosis (ADCP) and measured the ability of MAb 084-7D to cross-link CD16 and SARS-CoV-2 spike proteins expressed on cells as a proxy for antibody-dependent cellular cytotoxicity (ADCC). Both of these effector functions have been shown to contribute to decreased severity of SARS-CoV-2 infection and are required for optimal protection from infection by monoclonal antibodies (30). We compared MAb 084-7D to control MAbs, P2B-2F6 and CR3022. The 084-7D MAb mediated strong phagocytic activity against the Beta variant (AUC, 1,513) but up to 3-fold-weaker activity against the D614G and Omicron variants (AUCs, 209 and 468, respectively) (Fig. 3A). In comparison, P2B-2F6, which targets a class II RBD epitope, mediated strong ADCP against D614G but much lower levels against Beta and no activity against Omicron, as expected. The CR3022 MAb showed cross-reactive ADCP to all three variants, as previously reported (23). The 084-7D MAb exhibited ADCC activity against the Beta (AUC, 25,937) and Omicron (AUC, 15,551) variants, while no activity was detected for the D614G variant (Fig. 3B). The P2B-2F6 MAb displayed strong ADCC activity against the D614G variant (AUC, 113,631) as expected but no activity toward Beta and Omicron. CR3022 had similar activity across the three variants. Overall, the 084-7D MAb mediated Fc effector functionality with similar patterns of cross-reactivity to that observed in neutralization, exhibiting ADCP and ADCC activity against the Beta variant but reduced activity against Omicron.

### 084-7D is related to a broad and potent N417-dependent antibody isolated from early pandemic infection.

**FIG 4:**
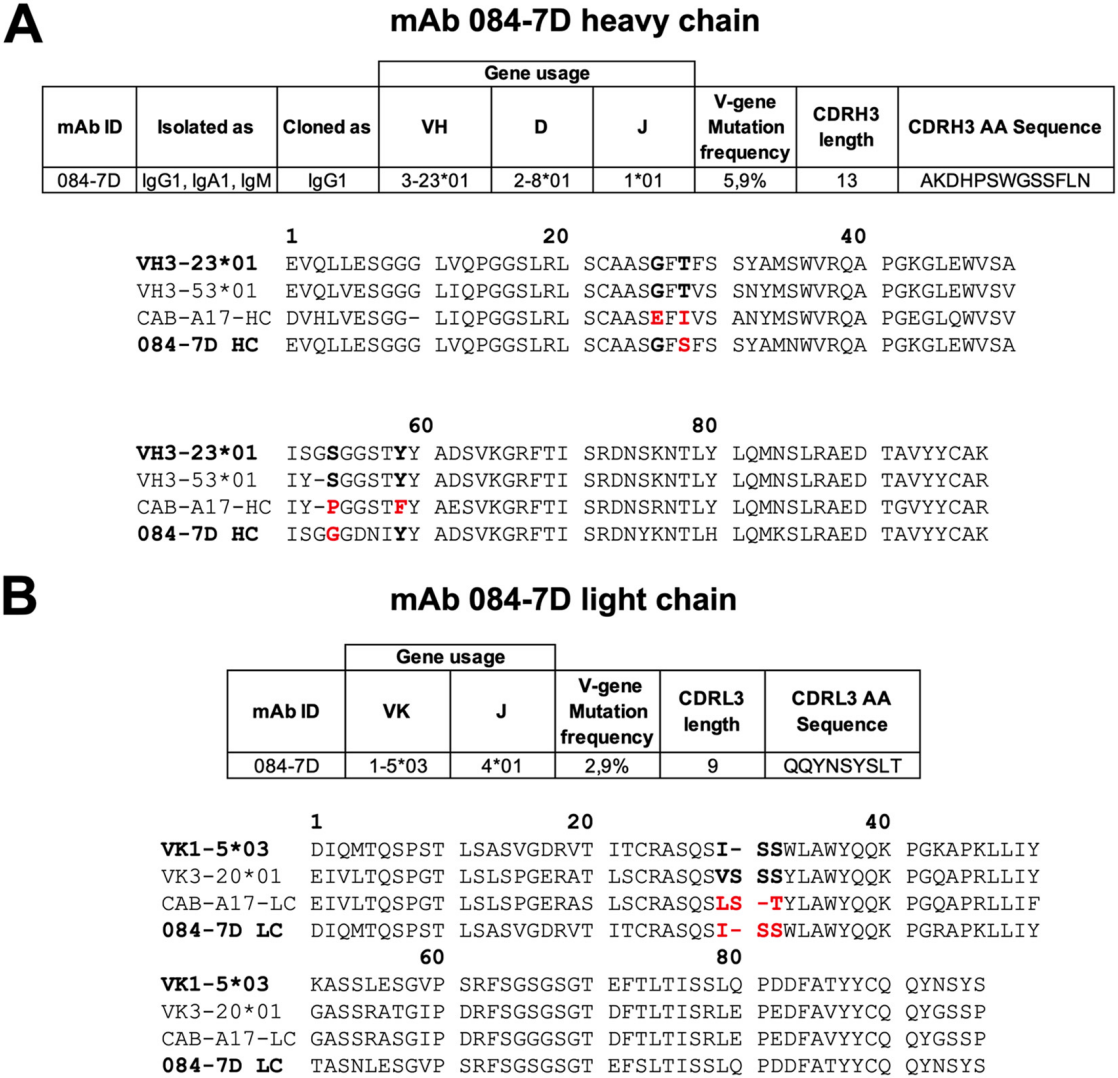
Genetic analysis of MAb 084-7D compared to a similar N417-dependent MAb. Heavy chain (A) and light chain (B) analysis of MAb 087-7D was conducted using IMGT/V-QUEST (https://www.imgt.org/IMGT_vquest/analysis). A multiple-sequence alignment of the germ line gene and MAb sequences for 084-7D and CAB-A17 was generated using ClustalW (https://www.genome.jp/tools-bin/clustalw). Key positions implicated in the development of breadth in the CAB-A17 MAb are shown in bold, and mutations from germ line gene residues in these regions are shown in red.

We next investigated the genetics of MAb 084-7D to determine whether it could be classified among other RBD-targeting MAbs based on germ line gene usage and epitope targeting (Fig. 4). The 084-7D antibody was isolated as an IgG1, IgA1, and IgM but was cloned and tested as an IgG1 for this study (Fig. 1A). The MAb uses the 3-23*01 variable heavy (VH) chain gene, exhibiting 5.9% somatic hypermutation in the variable gene region, despite being isolated early in infection (Fig. 4A). The 1-5*03 variable kappa (VK) chain gene is used by this MAb, and we observed less somatic hypermutation in the VK gene (2.9%) than for the heavy chain. We compared the 084-7D MAb to a previously reported SARS-CoV-2 RBD-specific antibody, CAB-A17, that utilizes the VH3-53*01 germ line gene, which is similar to VH3-23*01 (91% identity). The CAB-A17 lineage developed VOC cross-neutralization through interactions with the N417 residue, suggesting epitope targeting similar to that of MAb 084-7D (31).

Sheward et al. (31) identified four key mutations in the heavy chain of the broad MAb CAB-A17, which likely contributed to the increased breadth observed: G26E, T28I, S53P, and Y58F (Kabat numbering). Alignment of the VH3-23*01 (used by 084-7D), VH3-53*01 (used by CAB-A17), CAB-A17, and 084-7D heavy chain sequences (truncated at the CDRH3 region) showed that the 084-7D MAb had mutations at two of the same key residues, T28S and S53G, although the amino acid changes differed from the CAB-A17 MAb mutations (Fig. 4A).

Furthermore, the light chain of class I K417-targeting antibodies has been shown to play a role in epitope interactions through the CDRL1 ^28^VSSS^31^ motif (Kabat numbering) (14, 31). We noted that the 084-7D MAb possessed a germ line-encoded ISS motif, which is similar to the case with the CAB-A17 MAb, which also had a deletion in this region, resulting in an LST motif (Fig. 4B). Altogether, these data show that the 084-7D MAb is similar to a highly broad and potent MAb which targets a similar epitope.

## DISCUSSION

In this study, we investigated the cross-reactive potential of plasma elicited by three different variants that circulated in South Africa during three sequential waves of infection: the D614G, Beta, and Delta variants. Antibodies generated by the Beta and Delta variants exhibited higher levels of cross-reactivity than those triggered by the original D614G variant, confirming that SARS-CoV-2 variants elicit various antibody responses, potentially targeting different epitopes on the spike protein. Second, we used a MAb isolated from a Beta-infected donor to define an N417-dependent neutralizing antibody epitope shared between the Beta, Delta+, and Omicron variants. This MAb exhibited genetic features similar to those of a potent and broad N417-dependent antibody, suggesting that this site may be commonly targeted by SARS-CoV-2 cross-reactive MAbs.

The K417N mutation has been implicated in immune escape from Delta-elicited plasma, and this may partially explain the lack of cross-reactivity between Beta and Delta antibody responses (16). In line with this, we found that although Delta+ and Delta spike sequences only differ by one amino acid (N417 in Delta+ and K417 in Delta), antibodies elicited by the Beta variant showed cross-reactivity against the Delta+ VOI but very little cross-reactivity toward the Delta VOC. Our findings suggest that the Beta-elicited plasma responses we tested were preferentially targeting an epitope that was dependent on the N417 residue that is found in the Beta, Delta+ and Omicron VOCs. Our data confirm findings by other groups who have shown that the N417-directed response accounts for a large proportion of the Beta-elicited response in some individuals (27, 28).

In this study, we isolated an N417-dependent MAb from a Beta-infected individual which neutralized and performed Fc effector function against the Beta, Delta+, and Omicron VOCs. The 084-7D MAb utilizes the VH3-23*01 germ line gene, and members of this class of MAbs have previously been shown to target the RBD (32, 33). VH3 gene families are well documented in targeting this site, including class I antibodies which bind to the K417 residue (14, 32). The VH3 group of antibodies have been classed as public antibodies, suggesting that they can be easily elicited in SARS-CoV-2 infection and vaccination (32, 34, 35). Similar to the case with 084-7D MAb, low levels of somatic hypermutation are needed for most VH3 SARS-CoV-2 antibodies to neutralize their target, suggesting that germ line-encoded sequences, such as in CDRH2 and CDRL2, play a sufficient role in epitope recognition (14). These characteristics of MAb 084-7D make it attractive to pursue as a vaccine target.

Although many aspects of neutralization are germ line encoded, increased somatic hypermutation enhances the maturation of breadth to SARS-CoV-2 VOCs (36), as for other pathogens (37, 38). As MAb 084-7D was isolated from blood drawn only 2 days after the donor tested positive for SARS-CoV-2, this antibody was isolated too early to have accumulated substantial somatic hypermutation. Plasma from donor 084 collected at the same time point was highly potent against Omicron, but the 084-7D MAb displayed less potency against this variant. This suggests that there may be other antibodies in the plasma which are cross-reactive against Omicron.

The lack of neutralization and Fc effector of MAb 084-7D against Omicron compared to the Beta and Delta+ variants may be impacted by the fact that the Beta and Delta+ variants each possess three mutations in their RBDs, including the K417N mutations, while Omicron possesses at least 17 mutations in that region (5). The numerous mutations clustered around the Y501 residue in Omicron may have an impact on MAb 084-7D interactions, as this region has been shown to be important for class I antibody CDRL1 interactions (32). Although our data focused on the interaction between this MAb and the N417 residue, the complete epitope-paratope interaction must be investigated through the determination of a high-resolution structure. This will delineate the role of other residues in creating the full epitope of this MAb to decipher its relatively lower levels of potency toward Omicron.

A recently described MAb, CAB-A17, shares genetic and epitope-dependent traits similar to those of MAb 084-7D (31). However, MAb CAB-A17 has substantially more breadth across VOCs and is also more potent against Omicron than the 084-7D MAb (31). Exploring the differences between the two MAbs may suggest a mechanism required to achieve high levels of breadth and potency in this class of antibody. The CAB-A17 MAb contains key mutations that resulted in increased breadth toward the Omicron variant: G26E, T28I, S53P, and Y58F. The 084-7D MAb contains 2/4 of these mutations, although the residue changes differ: T28S and S53G. The changes in MAb CAB-A17 at these sites are much more substantial, especially the serine-to-proline change at position 53, which may alter the conformation of that region and therefore affect epitope binding. These differences may contribute to the disparity in breadth and potency between these two MAbs.

In addition to their distinct epitope-paratope interactions, the angle of approach is likely different between the two MAbs. CAB-A17 makes interactions with the N417 residue through the Y33 residue on the heavy chain (31). MAb 084-7D possesses an alanine at that position (A33) that would likely be unable to make strong contacts with the asparagine at 417 due to its shorter side chain. Therefore, it is plausible that MAb 084-7D interacts with the N417 residue using an alternative contact site(s) or binds through a different angle of approach. It is important to note that the evolution of the CAB-A17 MAb took place over 7 months, while 084-7D was isolated from acute infection. Tracking the evolution of MAb 084-7D will reveal whether somatic hypermutation led to increased potency and breadth and whether this maturation mimicked the pathway used by CAB-A17 or followed an independent route.

The identification of another novel N417-dependent MAb that can neutralize various VOCs/VOIs indicates that this epitope may be a good target for a cross-reactive response. However, we note that while we have extensive clinical follow-up, we cannot rule out the possibility that convalescent donors had experienced previous undocumented asymptomatic infection, which could alter the quality of humoral responses. Additionally, the use of patient samples from different hospitals during different waves raises the potential of systematic differences in patient characteristics that might affect cross-reactivity of serum. The CAB-A17 MAb was isolated earlier in the pandemic, presumably as a result of D614G infection, while the 084-7D MAb was isolated from Beta infection, again highlighting the universal targeting of this epitope by different variants. Despite the divergence of variants into “serotypes” or “clusters,” we show that the antibody response toward different variants can converge on shared epitopes. Finding MAbs that target conserved sites across variants will likely aid in the development of therapeutic MAb cocktails that can treat SARS-CoV-2 infection regardless of the infecting variant.

## MATERIALS AND METHODS

### Cohort description.

Plasma samples from the first SARS-CoV-2 wave (D614G infected) were obtained from a previously described cohort across various sites in South Africa prior to September 2020 (3). Second-wave samples (Beta infected) were obtained from a cohort of patients admitted to Groote Schuur Hospital, Cape Town, in December 2020 to January 2021 (19). Third-wave samples (Delta infected) were obtained from the Steve Biko Academic Hospital, Tshwane, from patients admitted in July 2021. In all waves, samples were collected when more than 90% of SARS-CoV-2 cases in South Africa were caused by the respective variants. Sequence confirmation was available for only a subset of samples, but all the samples that were sequenced corresponded to the appropriate variant for that wave. All samples were from HIV-uninfected individuals who were above 18 years of age and provided consent. Ethical clearance was obtained for each cohort from Human Research Ethics Committees from the University of Pretoria (247/2020) and University of Cape Town (R021/2020).

### SARS-CoV-2 antigen design and expression.

The pseudovirus SARS-CoV-2 Wuhan-1 spike was mutated using the QuikChange Lightning site-directed mutagenesis kit (Agilent Technologies) and NEBuilder HiFi DNA assembly master mix (New England Biolabs [NEB]) into the D614G wild type (614G WT) and the Beta (L18F, D80A, D215G, 242-244del, K417N, E484K, N501Y, D614G, and A701V), Delta (T19R, 156-157del, R158G, L452R, T478K, D614G, P681R, and D950N), Delta Plus (Delta+) (T19R, 156-157del, R158G, K417N, L452R, T478K, D614G, P681R, and D950N), Gamma (L18F, T20N, P26S, D138Y, R190S, K417T, E484K, N501Y, D614G, H655Y, T1027I, and V1176F), and Omicron (A67V, Δ69-70, T95I, G142D, Δ143-145, Δ211, L212I, 214EPE, G339D, S371L, S373P, S375F, K417N, N440K, G446S, S477N, T478K, E484A, Q493R, G496S, Q498R, N501Y, Y505H, T547K, D614G, H655Y, N679K, P681H, N764K, D796Y, N856K, Q954H, N969K, and L981F) variants. The SARS-CoV-1 spike was obtained from Genscript. Pseudoviruses were prepared as previously described (3). Briefly, human embryonic kidney (HEK) 293T cells were cotransfected with the SARS-CoV-2 spike plasmid of interest together with a firefly luciferase-encoding lentivirus backbone plasmid for 72 h. Culture supernatants were filter sterilized and stored at −70°C.

For soluble proteins, the SARS-CoV-2 D614G spike was obtained from Jason McLellan (University of Texas) and the SARS-CoV-2 RBD WT from Florian Krammer. The full spike proteins were mutated to produce the 614G WT and the Beta (L18F, D80A, D215G, 242-244del, K417N, E484K, N501Y, D614G, and A701V), Delta (T19R, 156-157del, R158G, L452R, T478K, D614G, P681R, and D950N), A.VOI.V2 (D80Y, DEL144, I210N, DELN211, D215G, R246M, DEL LAL247-249, W258L, R346K, T478R, E484K, H655Y, P681H, and Q957H), and Omicron (BA.1) (A67V, Δ69-70, T95I, G142D, Δ143-145, Δ211, L212I, 214EPE, G339D, S371L, S373P, S375F, K417N, N440K, G446S, S477N, T478K, E484A, Q493R, G496S, Q498R, N501Y, Y505H, T547K, D614G, H655Y, N679K, P681H, N764K, D796Y, N856K, Q954H, N969K, and L981F) spike proteins. SARS-CoV-2 subdomain 1 (SBD) protein (residues 321 to 591) and N-terminal domain (NTD) protein (residues 14 to 304) were designed in-house, ordered from Genscript, and cloned into a mammalian cell expression vector. Mutagenesis was used to produce SBD Beta (K417N, E484K, or N501Y) SBD K417N, SBD E484K, and SBD N501Y single mutants and NTD Beta (L18F, D80A, D215G, 242-244 del). Proteins were expressed in HEK 293F cells for 6 days, at 37°C, 70% humidity, and 10% CO_2_. Purification was carried out using a nickel-charged resin followed by size exclusion chromatography. Pure protein fractions were collected and frozen at −80°C until further use.

### Expression and purification of SARS-CoV-2-directed monoclonal antibodies.

Monoclonal antibodies were transfected in HEK 293F suspension cells using PEIMAX transfection reagent (Polysciences). Transfections were incubated at 220 rpm, 37°C, 70% humidity, and 10% CO_2_ for 6 to 7 days, and clarified supernatants were purified using protein A chromatography.

### SARS-CoV-2 monoclonal antibody isolation.

Cryopreserved PBMCs from a SARS-CoV-2-infected participant, SA-01-0084, were stained for SARS-CoV-2-specific B-cell markers before single-cell sorting. B cells marked as CD3^−^, CD14^−^, CD16^−^, CD19^+^, and Aqua vital dye negative were selected. Biotinylated SARS-CoV-2 Beta and D614G spike antigens were labeled with phycoerythrin (PE) and Alexa Fluor 647 (AF647), respectively, through the biotin-streptavidin interaction. Viable cells that bound the Beta and/or D614G spikes were single-cell sorted into 96-well plates, and heavy and light chain genes were PCR amplified.

### Single-cell PCR amplification of heavy and light chain variable genes.

Genes encoding immunoglobulin heavy chain variable region (IGHV) and immunoglobulin λ light chain variable region (IGLV) chains were amplified from sorted cells by reverse real-time and nested PCR. Reverse transcription was performed using Superscript III reverse transcriptase (Invitrogen) and random hexamer primers (39, 40). Following cDNA synthesis, VH, Vκ, and Vλ antibody genes were amplified as previously described (41, 42). Determination of gene usage was done using IMGT/V-QUEST (https://www.imgt.org/IMGT_vquest/analysis).

### Pseudovirus-based neutralization assay.

Heat-inactivated plasma samples from COVID-19 convalescent donors or MAbs were incubated with the SARS-CoV-2 pseudoviruses for 1 h at 37°C and 5% CO_2_. Subsequently, 1 × 10^4^ HEK 293T cells engineered to overexpress ACE-2, provided by Michael Farzan, were added and incubated at 37°C and 5% CO_2_ for 72 h, after which the luminescence of the luciferase gene was measured. Monoclonal antibodies CB6, CA1, and CR3022 were used as controls.

### SARS-CoV-2 enzyme-linked immunosorbent assay (ELISA).

Ninety-six-well high-binding plates with 2 μg/mL of the respective antigen were incubated overnight at 4°C. After blocking with 5% milk powder in 1× phosphate-buffered saline (PBS) and 0.05% Tween 20, MAbs at a 5-μg/mL starting concentration was serially diluted and incubated at room temperature for 1.5 h. Subsequent washing was followed by the addition of an anti-human horseradish peroxidase-conjugated secondary antibody diluted 1:3,000, and the plates were incubated for a further 1 h. Tetramethyl benzidine (TMB) substrate (Thermo Fisher Scientific) was added, followed by 1 M H_2_SO_4_ to stop the reaction, with absorbance of the reaction measured at an optical density of 450 nm. MAbs CR3022, P2B-2FB, and palivizumab served as controls.

### ADCC assay.

The ability of the MAb to cross-link between FcγRIIIa (CD16) and spike expressed on cells was used as a proxy for antibody-dependent cellular cytotoxicity (ADCC). To express cell surface spike, HEK 293T cells were transfected with SARS-CoV-2 614G WT-, Beta-, or Omicron-expressing plasmids, with incubation at 37°C over 2 days. Spike-expressing cells were incubated with 100 μg/mL of 084-7D or control MAbs titrated by four dilutions of 1 in 5 in RPMI medium, 10% fetal bovine serum (FBS), and 1% penicillin-streptomycin for an hour at 37°C. Following this, Jurkat-Lucia NFAT-CD16 cells (Invitrogen) were added to the reaction and incubated for a further 24 h at 37°C with 10% CO_2_. Signal was read on a luminometer by adding 20 μL of supernatant and 50 μL of QUANTI-Luc secreted luciferase to white 96-well plates. CR3022, P2B-2F6, and palivizumab served as controls, and data across spikes were normalized using CR3022.

### Antibody-dependent cellular phagocytosis (ADCP) assay.

Biotinylated SARS-CoV-2 614G WT, Beta, and Omicron spike proteins were used to coat beads by incubating fluorescent neutravidin beads with 10 μg/mL of antibody for 2 h as described elsewhere (22). Overnight incubation was carried out with monocytic THP-1 cells, followed by analysis on a FACSAria II instrument (BD Biosciences). The percentage of engulfed fluorescent beads within the THP-1 cells multiplied by the geometric mean was calculated, and a final phagocytosis score was determined by subtracting the fluorescence score of the no-antibody control. CR3022, P2B-2F6, and palivizumab served as controls.

## References

[1] Hirabara SM, Serdan TD, Gorjao R, Masi LN, Pithon-Curi TC, Covas DT, Curi R, Durigon EL. 2022. SARS-COV-2 variants: differences and potential of immune evasion. Front Cell Infect Microbiol 11:1401. 10.3389/fcimb.2021.781429. PMC8805732 35118007

[2] Tegally H, Wilkinson E, Giovanetti M, Iranzadeh A, Fonseca V, Giandhari J, Doolabh D, Pillay S, San EJ, Msomi N, Mlisana K, von Gottberg A, Walaza S, Allam M, Ismail A, Mohale T, Glass AJ, Engelbrecht S, Van Zyl G, Preiser W, Petruccione F, Sigal A, Hardie D, Marais G, Hsiao N-Y, Korsman S, Davies M-A, Tyers L, Mudau I, York D, Maslo C, Goedhals D, Abrahams S, Laguda-Akingba O, Alisoltani-Dehkordi A, Godzik A, Wibmer CK, Sewell BT, Lourenço J, Alcantara LCJ, Kosakovsky Pond SL, Weaver S, Martin D, Lessells RJ, Bhiman JN, Williamson C, de Oliveira T. 2021. Detection of a SARS-CoV-2 variant of concern in South Africa. Nature 592:438–443. 10.1038/s41586-021-03402-9. 33690265

[3] Wibmer CK, Ayres F, Hermanus T, Madzivhandila M, Kgagudi P, Oosthuysen B, Lambson BE, de Oliveira T, Vermeulen M, van der Berg K, Rossouw T, Boswell M, Ueckermann V, Meiring S, von Gottberg A, Cohen C, Morris L, Bhiman JN, Moore PL. 2021. SARS-CoV-2 501Y.V2 escapes neutralization by South African COVID-19 donor plasma. Nat Med 27:622–625. 10.1038/s41591-021-01285-x. 33654292

[4] Scheepers C, Everatt J, Amoako DG, Tegally H, Wibmer CK, Mnguni A, Ismail A, Mahlangu B, Lambson BE, Richardson SI, Martin DP, Wilkinson E, San JE, Giandhari J, Manamela N, Ntuli N, Kgagudi P, Cele S, Pillay S, Mohale T, Ramphal U, Naidoo Y, Khumalo ZT, Kwatra G, Gray G, Bekker L-G, Madhi SA, Baillie V, Van Voorhis WC, Treurnicht FK, Venter M, Mlisana K, Wolter N, Sigal A, Williamson C, Hsiao N-Y, Msomi N, Maponga T, Preiser W, Makatini Z, Lessells R, Moore PL, de Oliveira T, von Gottberg A, Bhiman JN, NGS-SA. 2021. Emergence and phenotypic characterization of C.1.2, a globally detected lineage that rapidly accumulated mutations of concern. medRxiv. 10.1101/2021.08.20.21262342.

[5] Viana R, Moyo S, Amoako DG, Tegally H, Scheepers C, Althaus CL, Anyaneji UJ, Bester PA, Boni MF, Chand M, Choga WT, Colquhoun R, Davids M, Deforche K, Doolabh D, Du Plessis L, Engelbrecht S, Everatt J, Giandhari J, Giovanetti M, Hardie D, Hill V, Hsiao N-Y, Iranzadeh A, Ismail A, Joseph C, Joseph R, Koopile L, Kosakovsky Pond SL, Kraemer MUG, Kuate-Lere L, Laguda-Akingba O, Lesetedi-Mafoko O, Lessells RJ, Lockman S, Lucaci AG, Maharaj A, Mahlangu B, Maponga T, Mahlakwane K, Makatini Z, Marais G, Maruapula D, Masupu K, Matshaba M, Mayaphi S, Mbhele N, Mbulawa MB, Mendes A, Mlisana K, et al. 2022. Rapid epidemic expansion of the SARS-CoV-2 Omicron variant in southern Africa. Nature 603:679–686. 10.1038/d41586-021-03832-5. 35042229 PMC8942855

[6] de Oliveira T, Lutucuta S, Nkengasong J, Morais J, Paixão JP, Neto Z, Afonso P, Miranda J, David K, Inglês L, Raisa Rivas Carralero APAP, Freitas HR, Mufinda F, Tessema SK, Tegally H, San EJ, Wilkinson E, Giandhari J, Pillay S, Giovanetti M, Naidoo Y, Katzourakis A, Ghafari M, Singh L, Tshiabuila D, Martin D, Lessells RJ. 2021. A novel variant of interest of SARS-CoV-2 with multiple spike mutations detected through travel surveillance in Africa. medRxiv. 10.1101/2021.03.30.21254323.

[7] Sheward DJ, Kim C, Ehling RA, Pankow A, Dopico XC, Martin D, Reddy S, Dillner J, Karlsson Hedestam GB, Albert J, Murrell B. 2021. Variable loss of antibody potency against SARS-CoV-2 B.1.1.529 (Omicron). bioRxiv. 10.1101/2021.12.19.473354.

[8] Cele S, Jackson L, Khoury DS, Khan K, Moyo-Gwete T, Tegally H, San JE, Cromer D, Scheepers C, Amoako DG, Karim F, Bernstein M, Lustig G, Archary D, Smith M, Ganga Y, Jule Z, Reedoy K, Hwa S-H, Giandhari J, Blackburn JM, Gosnell BI, Abdool Karim SS, Hanekom W, Davies M-A, Hsiao M, Martin D, Mlisana K, Wibmer CK, Williamson C, York D, Harrichandparsad R, Herbst K, Jeena P, Khoza T, Kløverpris H, Leslie A, Madansein R, Magula N, Manickchund N, Marakalala M, Mazibuko M, Moshabela M, Mthabela N, Naidoo K, Ndhlovu Z, Ndung’u T, Ngcobo N, Nyamande K, Patel V, NGS-SA, et al. 2022. Omicron extensively but incompletely escapes Pfizer BNT162b2 neutralization. Nature 602:654–656. 10.1038/d41586-021-03824-5. 35016196 PMC8866126

[9] Garcia-Beltran WF, St. Denis KJ, Hoelzemer A, Lam EC, Nitido AD, Sheehan ML, Berrios C, Ofoman O, Chang CC, Hauser BM, Feldman J, Roederer AL, Gregory DJ, Poznansky MC, Schmidt AG, Iafrate AJ, Naranbhai V, Balazs AB. 2022. mRNA-based COVID-19 vaccine boosters induce neutralizing immunity against SARS-CoV-2 Omicron variant. Cell 185:457–466.e4. 10.1016/j.cell.2021.12.033. 34995482 PMC8733787

[10] Laurini E, Marson D, Aulic S, Fermeglia A, Pricl S. 2021. Molecular rationale for SARS-CoV-2 spike circulating mutations able to escape bamlanivimab and etesevimab monoclonal antibodies. Sci Rep 11:20274. 10.1038/s41598-021-99827-3. 34642465 PMC8511038

[11] Arora P, Kempf A, Nehlmeier I, Graichen L, Sidarovich A, Winkler MS, Schulz S, Jäck H-M, Stankov MV, Behrens GMN, Pöhlmann S, Hoffmann M. 2021. Delta variant (B.1.617.2) sublineages do not show increased neutralization resistance. Cell Mol Immunol 18:2557–2559. 10.1038/s41423-021-00772-y. 34635807 PMC8503871

[12] Kumar S, Karuppanan K, Subramaniam G. 2022. Omicron (BA.1) and sub-variants (BA.1, BA.2 and BA.3) of SARS-CoV-2 spike infectivity and pathogenicity: a comparative sequence and structural-based computational assessment. bioRxiv. 10.1101/2022.02.11.480029. PMC9347785 35680610

[13] Martin DP, Weaver S, Tegally H, San JE, Shank SD, Wilkinson E, Lucaci AG, Giandhari J, Naidoo S, Pillay Y, Singh L, Lessells RJ, Gupta RK, Wertheim JO, Nekturenko A, Murrell B, Harkins GW, Lemey P, MacLean OA, Robertson DL, de Oliveira T, Kosakovsky Pond SL, COVID-19 Genomics UK (COG-UK). 2021. The emergence and ongoing convergent evolution of the SARS-CoV-2 N501Y lineages. Cell 184:5189–5200.e7. 10.1016/j.cell.2021.09.003. 34537136 PMC8421097

[14] Barnes CO, Jette CA, Abernathy ME, Dam K-MA, Esswein SR, Gristick HB, Malyutin AG, Sharaf NG, Huey-Tubman KE, Lee YE, Robbiani DF, Nussenzweig MC, West AP, Bjorkman PJ. 2020. SARS-CoV-2 neutralizing antibody structures inform therapeutic strategies. Nature 588:682–686. 10.1038/s41586-020-2852-1. 33045718 PMC8092461

[15] Greaney AJ, Starr TN, Eguia RT, Loes AN, Khan K, Karim F, Cele S, Bowen JE, Logue JK, Corti D, Veesler D, Chu HY, Sigal A, Bloom JD. 2022. A SARS-CoV-2 variant elicits an antibody response with a shifted immunodominance hierarchy. PLoS Pathog 18:e1010248. 10.1371/journal.ppat.1010248. 35134084 PMC8856557

[16] Greaney AJ, Eguia RT, Starr TN, Khan K, Franko N, Logue JK, Lord SM, Speake C, Chu HY, Sigal A, Bloom JD. 2022. The SARS-CoV-2 Delta variant induces an antibody response largely focused on class 1 and 2 antibody epitopes. bioRxiv. 10.1101/2022.03.12.484088. PMC9275729 35767821

[17] Piccoli L, Park Y-J, Tortorici MA, Czudnochowski N, Walls AC, Beltramello M, Silacci-Fregni C, Pinto D, Rosen LE, Bowen JE, Acton OJ, Jaconi S, Guarino B, Minola A, Zatta F, Sprugasci N, Bassi J, Peter A, De Marco A, Nix JC, Mele F, Jovic S, Rodriguez BF, Gupta SV, Jin F, Piumatti G, Lo Presti G, Pellanda AF, Biggiogero M, Tarkowski M, Pizzuto MS, Cameroni E, Havenar-Daughton C, Smithey M, Hong D, Lepori V, Albanese E, Ceschi A, Bernasconi E, Elzi L, Ferrari P, Garzoni C, Riva A, Snell G, Sallusto F, Fink K, Virgin HW, Lanzavecchia A, Corti D, Veesler D. 2020. Mapping neutralizing and immunodominant sites on the SARS-CoV-2 spike receptor-binding domain by structure-guided high-resolution serology. Cell 183:1024–1042.e21. 10.1016/j.cell.2020.09.037. 32991844 PMC7494283

[18] Greaney AJ, Loes AN, Gentles LE, Crawford KH, Starr TN, Malone KD, Chu HY, Bloom JD. 2021. Antibodies elicited by mRNA-1273 vaccination bind more broadly to the receptor binding domain than do those from SARS-CoV-2 infection. Sci Transl Med 13:eabi9915. 10.1126/scitranslmed.abi9915. 34103407 PMC8369496

[19] Moyo-Gwete T, Madzivhandila M, Makhado Z, Ayres F, Mhlanga D, Oosthuysen B, Lambson BE, Kgagudi P, Tegally H, Iranzadeh A, Doolabh D, Tyers L, Chinhoyi LR, Mennen M, Skelem S, Marais G, Wibmer CK, Bhiman JN, Ueckermann V, Rossouw T, Boswell M, de Oliveira T, Williamson C, Burgers WA, Ntusi N, Morris L, Moore PL. 2021. Cross-reactive neutralizing antibody responses elicited by SARS-CoV-2 501Y.V2 (B.1.351). N Engl J Med 384:2161–2163. 10.1056/NEJMc2104192. 33826816 PMC8063886

[20] Cele S, Karim F, Lustig G, San JE, Hermanus T, Tegally H, Snyman J, Moyo-Gwete T, Wilkinson E, Bernstein M, Khan K, Hwa S-H, Tilles SW, Singh L, Giandhari J, Mthabela N, Mazibuko M, Ganga Y, Gosnell BI, Karim SSA, Hanekom W, Van Voorhis WC, Ndung’u T, Lessells RJ, Moore PL, Moosa M-YS, de Oliveira T, Sigal A, COMMIT-KZN Team. 2022. SARS-CoV-2 prolonged infection during advanced HIV disease evolves extensive immune escape. Cell Host Microbe 30:154–162.e5. 10.1016/j.chom.2022.01.005. 35120605 PMC8758318

[21] Liu C, Ginn HM, Dejnirattisai W, Supasa P, Wang B, Tuekprakhon A, Nutalai R, Zhou D, Mentzer AJ, Zhao Y, Duyvesteyn HME, López-Camacho C, Slon-Campos J, Walter TS, Skelly D, Johnson SA, Ritter TG, Mason C, Costa Clemens SA, Gomes Naveca F, Nascimento V, Nascimento F, Fernandes da Costa C, Resende PC, Pauvolid-Correa A, Siqueira MM, Dold C, Temperton N, Dong T, Pollard AJ, Knight JC, Crook D, Lambe T, Clutterbuck E, Bibi S, Flaxman A, Bittaye M, Belij-Rammerstorfer S, Gilbert SC, Malik T, Carroll MW, Klenerman P, Barnes E, Dunachie SJ, Baillie V, Serafin N, Ditse Z, Da Silva K, Paterson NG, Williams MA, et al. 2021. Reduced neutralization of SARS-CoV-2 B.1.617 by vaccine and convalescent serum. Cell 184:4220–4236.e13. 10.1016/j.cell.2021.06.020. 34242578 PMC8218332

[22] Dupont L, Snell LB, Graham C, Seow J, Merrick B, Lechmere T, Maguire TJA, Hallett SR, Pickering S, Charalampous T, Alcolea-Medina A, Huettner I, Jimenez-Guardeño JM, Acors S, Almeida N, Cox D, Dickenson RE, Galao RP, Kouphou N, Lista MJ, Ortega-Prieto AM, Wilson H, Winstone H, Fairhead C, Su JZ, Nebbia G, Batra R, Neil S, Shankar-Hari M, Edgeworth JD, Malim MH, Doores KJ. 2021. Neutralizing antibody activity in convalescent sera from infection in humans with SARS-CoV-2 and variants of concern. Nat Microbiol 6:1433–1442. 10.1038/s41564-021-00974-0. 34654917 PMC8556155

[23] Richardson SI, Madzorera VS, Spencer H, Manamela NP, van der Mescht MA, Lambson BE, Oosthuysen B, Ayres F, Makhado Z, Moyo-Gwete T, Mzindle N, Motlou T, Strydom A, Mendes A, Tegally H, de Beer Z, Roma de Villiers T, Bodenstein A, van den Berg G, Venter M, de Oliviera T, Ueckermann V, Rossouw TM, Boswell MT, Moore PL. 2022. SARS-CoV-2 Omicron triggers cross-reactive neutralization and Fc effector functions in previously vaccinated, but not unvaccinated individuals. Cell Host Microbe 30:880–886.e4. 10.1016/j.chom.2022.03.029. 35436444 PMC8947963

[24] Eguia RT, Crawford KHD, Stevens-Ayers T, Kelnhofer-Millevolte L, Greninger AL, Englund JA, Boeckh MJ, Bloom JD. 2021. A human coronavirus evolves antigenically to escape antibody immunity. PLoS Pathog 17:e1009453. 10.1371/journal.ppat.1009453. 33831132 PMC8031418

[25] Teyssou E, Delagrèverie H, Visseaux B, Lambert-Niclot S, Brichler S, Ferre V, Marot S, Jary A, Todesco E, Schnuriger A, Ghidaoui E, Abdi B, Akhavan S, Houhou-Fidouh N, Charpentier C, Morand-Joubert L, Boutolleau D, Descamps D, Calvez V, Marcelin AG, Soulie C. 2021. The Delta SARS-CoV-2 variant has a higher viral load than the Beta and the historical variants in nasopharyngeal samples from newly diagnosed COVID-19 patients. J Infect 83:e1–e3. 10.1016/j.jinf.2021.08.027. PMC8375250 34419559

[26] van der Straten K, Guerra D, van Gils MJ, Bontjer I, Caniels TG, van Willigen HDG, Wynberg E, Poniman M, Burger JA, Bouhuijs JH, van Rijswijk J, Olijhoek W, Liesdek MH, Lavell AHA, Appelman B, Sikkens JJ, Bomers MK, Han AX, Nichols BE, Prins M, Vennema H, Reusken C, de Jong MD, de Bree GJ, Russell CA, Eggink D, Sanders RW. 2022. Mapping the antigenic diversification of SARS-CoV-2. medRxiv. 10.1101/2022.01.03.21268582.

[27] Wilks SH, Mühlemann B, Shen X, Türeli S, LeGresley EB, Netzl A, Caniza MA, Chacaltana-Huarcaya JN, Daniell X, Datto MB, Denny TN, Drosten C, Fouchier RAM, Garcia PJ, Halfmann PJ, Jassem A, Jones TC, Kawaoka Y, Krammer F, McDanal C, Pajon R, Simon V, Stockwell M, Tang H, van Bakel H, Webby R, Montefiori DC, Smith DJ. 2022. Mapping SARS-CoV-2 antigenic relationships and serological responses. bioRxiv. 10.1101/2022.01.28.477987. PMC12145880 37797027

[28] Reincke SM, Yuan M, Kornau H-C, Corman VM, van Hoof S, Sánchez-Sendin E, Ramberger M, Yu W, Hua Y, Tien H, Schmidt ML, Schwarz T, Jeworowski LM, Brandl SE, Rasmussen HF, Homeyer MA, Stöffler L, Barner M, Kunkel D, Huo S, Horler J, von Wardenburg N, Kroidl I, Eser TM, Wieser A, Geldmacher C, Hoelscher M, Gänzer H, Weiss G, Schmitz D, Drosten C, Prüss H, Wilson IA, Kreye J. 2022. SARS-CoV-2 Beta variant infection elicits potent lineage-specific and cross-reactive antibodies. Science 375:782–787. 10.1126/science.abm5835. 35076281 PMC8939768

[29] Richardson SI, Manamela NP, Motsoeneng BM, Kaldine H, Ayres F, Makhado Z, Mennen M, Skelem S, Williams N, Sullivan NJ, Misasi J, Gray GG, Bekker L-G, Ueckermann V, Rossouw TM, Boswell MT, Ntusi NAB, Burgers WA, Moore PL. 2022. SARS-CoV-2 Beta and Delta variants trigger Fc effector function with increased cross-reactivity. Cell Rep Med 3:100510. 10.1016/j.xcrm.2022.100510. 35233544 PMC8761540

[30] Zohar T, Loos C, Fischinger S, Atyeo C, Wang C, Slein MD, Burke J, Yu J, Feldman J, Hauser BM, Caradonna T, Schmidt AG, Cai Y, Streeck H, Ryan ET, Barouch DH, Charles RC, Lauffenburger DA, Alter G. 2020. Compromised humoral functional evolution tracks with SARS-CoV-2 mortality. Cell 183:1508–1519.e12. 10.1016/j.cell.2020.10.052. 33207184 PMC7608014

[31] Sheward DJ, Pushparaj P, Das H, Kim C, Kim S, Hanke L, Dyrdak R, McInerney GM, Albert J, Murrell B. 2022. Structural basis of Omicron neutralization by affinity-matured public antibodies. bioRxiv. 10.1101/2022.01.03.474825. PMC11228396 38761799

[32] Yuan M, Liu H, Wu NC, Lee C-CD, Zhu X, Zhao F, Huang D, Yu W, Hua Y, Tien H, Rogers TF, Landais E, Sok D, Jardine JG, Burton DR, Wilson IA. 2020. Structural basis of a shared antibody response to SARS-CoV-2. Science 369:1119–1123. 10.1126/science.abd2321. 32661058 PMC7402627

[33] Ju B, Zhang Q, Ge J, Wang R, Sun J, Ge X, Yu J, Shan S, Zhou B, Song S, Tang X, Yu J, Lan J, Yuan J, Wang H, Zhao J, Zhang S, Wang Y, Shi X, Liu L, Zhao J, Wang X, Zhang Z, Zhang L. 2020. Human neutralizing antibodies elicited by SARS-CoV-2 infection. Nature 584:115–119. 10.1038/s41586-020-2380-z. 32454513

[34] Xu H, Wang B, Zhao T-N, Liang Z-T, Peng T-B, Song X-H, Wu J-J, Wang Y-C, Su X-D. 2021. Structure-based analyses of neutralization antibodies interacting with naturally occurring SARS-CoV-2 RBD variants. Cell Res 31:1126–1129. 10.1038/s41422-021-00554-1. 34480123 PMC8413711

[35] Cao Y, Su B, Guo X, Sun W, Deng Y, Bao L, Zhu Q, Zhang X, Zheng Y, Geng C, Chai X, He R, Li X, Lv Q, Zhu H, Deng W, Xu Y, Wang Y, Qiao L, Tan Y, Song L, Wang G, Du X, Gao N, Liu J, Xiao J, Su X-D, Du Z, Feng Y, Qin C, Qin C, Jin R, Xie XS. 2020. Potent neutralizing antibodies against SARS-CoV-2 identified by high-throughput single-cell sequencing of convalescent patients’ B cells. Cell 182:73–84.e16. 10.1016/j.cell.2020.05.025. 32425270 PMC7231725

[36] Muecksch F, Weisblum Y, Barnes CO, Schmidt F, Schaefer-Babajew D, Wang Z, Lorenzi JCC, Flyak AI, DeLaitsch AT, Huey-Tubman KE, Hou S, Schiffer CA, Gaebler C, Da Silva J, Poston D, Finkin S, Cho A, Cipolla M, Oliveira TY, Millard KG, Ramos V, Gazumyan A, Rutkowska M, Caskey M, Nussenzweig MC, Bjorkman PJ, Hatziioannou T, Bieniasz PD. 2021. Affinity maturation of SARS-CoV-2 neutralizing antibodies confers potency, breadth, and resilience to viral escape mutations. Immunity 54:1853–1868.e7. 10.1016/j.immuni.2021.07.008. 34331873 PMC8323339

[37] Derdeyn CA, Moore PL, Morris L. 2014. Development of broadly neutralizing antibodies from autologous neutralizing antibody responses in HIV infection. Curr Opin HIV AIDS 9:210–216. 10.1097/COH.0000000000000057. 24662931 PMC4068799

[38] Klein F, Diskin R, Scheid JF, Gaebler C, Mouquet H, Georgiev IS, Pancera M, Zhou T, Incesu RB, Fu BZ, Gnanapragasam PN, Oliveira TY, Seaman MS, Kwong PD, Bjorkman PJ, Nussenzweig MC. 2013. Somatic mutations of the immunoglobulin framework are generally required for broad and potent HIV-1 neutralization. Cell 153:126–138. 10.1016/j.cell.2013.03.018. 23540694 PMC3792590

[39] Tiller T, Meffre E, Yurasov S, Tsuiji M, Nussenzweig MC, Wardemann H. 2008. Efficient generation of monoclonal antibodies from single human B cells by single cell RT-PCR and expression vector cloning. J Immunol Methods 329:112–124. 10.1016/j.jim.2007.09.017. 17996249 PMC2243222

[40] Scheid JF, Mouquet H, Ueberheide B, Diskin R, Klein F, Oliveira TYK, Pietzsch J, Fenyo D, Abadir A, Velinzon K, Hurley A, Myung S, Boulad F, Poignard P, Burton DR, Pereyra F, Ho DD, Walker BD, Seaman MS, Bjorkman PJ, Chait BT, Nussenzweig MC. 2011. Sequence and structural convergence of broad and potent HIV antibodies that mimic CD4 binding. Science 333:1633–1637. 10.1126/science.1207227. 21764753 PMC3351836

[41] Doria-Rose NA, Bhiman JN, Roark RS, Schramm CA, Gorman J, Chuang GY, Pancera M, Cale EM, Ernandes MJ, Louder MK, Asokan M, Bailer RT, Druz A, Fraschilla IR, Garrett NJ, Jarosinski M, Lynch RM, McKee K, O’Dell S, Pegu A, Schmidt SD, Staupe RP, Sutton MS, Wang K, Wibmer CK, Haynes BF, Abdool-Karim S, Shapiro L, Kwong PD, Moore PL, Morris L, Mascola JR. 2016. New member of the V1V2-directed CAP256-VRC26 lineage that shows increased breadth and exceptional potency. J Virol 90:76–91. 10.1128/JVI.01791-15. 26468542 PMC4702551

[42] Liao H-X, Levesque MC, Nagel A, Dixon A, Zhang R, Walter E, Parks R, Whitesides J, Marshall DJ, Hwang K-K, Yang Y, Chen X, Gao F, Munshaw S, Kepler TB, Denny T, Moody MA, Haynes BF. 2009. High-throughput isolation of immunoglobulin genes from single human B cells and expression as monoclonal antibodies. J Virol Methods 158:171–179. 10.1016/j.jviromet.2009.02.014. 19428587 PMC2805188

